# Advanced Care Planning for Hospitalized Patients Following Clinician Notification of Patient Mortality by a Machine Learning Algorithm

**DOI:** 10.1001/jamanetworkopen.2023.8795

**Published:** 2023-04-18

**Authors:** Stephen Chi, Seunghwan Kim, Matthew Reuter, Katharine Ponzillo, Debra Parker Oliver, Randi Foraker, Kevin Heard, Jingxia Liu, Kyle Pitzer, Patrick White, Nathan Moore

**Affiliations:** 1 Division of Pulmonary and Critical Care Medicine, Washington University in St Louis, St Louis, Missouri; 2 Institute for Informatics, Washington University in St Louis, St Louis, Missouri; 3 BJC Medical Group, St Louis, Missouri; 4 Division of Palliative Medicine, Department of Medicine, Washington University in St Louis, St Louis, Missouri; 5 Division of Public Health Sciences, Department of Surgery, Washington University in St Louis, St Louis, Missouri; 6 Division of Biostatistics, Washington University in St Louis, St Louis, Missouri

## Abstract

**Importance:**

Goal-concordant care is an ongoing challenge in hospital settings. Identification of high mortality risk within 30 days may call attention to the need to have serious illness conversations, including the documentation of patient goals of care.

**Objective:**

To examine goals of care discussions (GOCDs) in a community hospital setting with patients identified as having a high risk of mortality by a machine learning mortality prediction algorithm.

**Design, Setting, and Participants:**

This cohort study took place at community hospitals within 1 health care system. Participants included adult patients with a high risk of 30-day mortality who were admitted to 1 of 4 hospitals between January 2 and July 15, 2021. Patient encounters of inpatients in the intervention hospital where physicians were notified of the computed high risk mortality score were compared with patient encounters of inpatients in 3 community hospitals without the intervention (ie, matched control).

**Intervention:**

Physicians of patients with a high risk of mortality within 30 days received notification and were encouraged to arrange for GOCDs.

**Main Outcomes and Measures:**

The primary outcome was the percentage change of documented GOCDs prior to discharge. Propensity-score matching was completed on a preintervention and postintervention period using age, sex, race, COVID-19 status, and machine learning-predicted mortality risk scores. A difference-in-difference analysis validated the results.

**Results:**

Overall, 537 patients were included in this study with 201 in the preintervention period (94 in the intervention group; 104 in the control group) and 336 patients in the postintervention period. The intervention and control groups included 168 patients per group and were well-balanced in age (mean [SD], 79.3 [9.60] vs 79.6 [9.21] years; standardized mean difference [SMD], 0.03), sex (female, 85 [51%] vs 85 [51%]; SMD, 0), race (White patients, 145 [86%] vs 144 [86%]; SMD 0.006), and Charlson comorbidities (median [range], 8.00 [2.00-15.0] vs 9.00 [2.00 to 19.0]; SMD, 0.34). Patients in the intervention group from preintervention to postintervention period were associated with being 5 times more likely to have documented GOCDs (OR, 5.11 [95% CI, 1.93 to 13.42]; *P* = .001) by discharge compared with matched controls, and GOCD occurred significantly earlier in the hospitalization in the intervention patients as compared with matched controls (median, 4 [95% CI, 3 to 6] days vs 16 [95% CI, 15 to not applicable] days; *P* < .001). Similar findings were observed for Black patient and White patient subgroups.

**Conclusions and Relevance:**

In this cohort study, patients whose physicians had knowledge of high-risk predictions from machine learning mortality algorithms were associated with being 5 times more likely to have documented GOCDs than matched controls. Additional external validation is needed to determine if similar interventions would be helpful at other institutions.

## Key Points

**Question:**

Is informing physicians of a patient’s high risk of mortality (within 30 days), as identified by a machine learning mortality prediction algorithm, associated with a documented goals of care discussion (GOCD) before discharge?

**Findings:**

In this cohort study with 537 participants, patients in the intervention group from the preintervention to postintervention period were associated with being 5 times more likely to have documented GOCD compared with matched controls.

**Meaning:**

These findings suggest that physicians with data about high mortality risk for individual patients are more likely to have documented GOCDs.

## Teaser

This cohort study examined whether informing physicians of a patient’s high risk of mortality was associated with changes in the likelihood of documented goals of care discussion before discharge.

## Introduction

The delivery of goal-concordant care to hospitalized patients with serious and life-limiting illnesses remains a clinical challenge. While many patients may prioritize comfort more than the prolongation of life, patients frequently receive aggressive, intensive, and potentially futile care in the days and weeks prior to death.^1,2,3,4,5^ Serious illness communication (SIC) helps patients with serious illness and their clinicians engage in dialogue regarding their goals, values, and priorities to help enhance goal-concordant care.^6^ Goals of care discussions (GOCDs) use a patient’s underlying values and priorities, established within the existing clinical context, to guide decisions about the use of or limitations of specific medical interventions.^7,8^ In the inpatient setting, trials promoting SIC have shown promising results, including increased GOCD documentation, decreased intensive care unit (ICU) transfers and health care costs, and timelier palliative care and hospice referrals.^9,10,11,12,13,14,15^ Yet, GOCDs have been met with resistance within clinical practice due to multiple logistical and practical barriers.^16,17,18,19,20^

Among the implementation barriers to these conversations are limited clinician time and difficulty identifying appropriate patients. Targeting GOCDs based on patient mortality risk has the potential to address both these deficiencies. A range of inpatient interventions have been trialed to that effect, from automated clinician notifications to opt-out palliative care consultations, with results generally showing increases in goals of care and advanced care planning (ACP) discussions and decreases in health care utilization.^15,21,22,23,24^ These studies have significant heterogeneity in patient selection and implementation, partly from the historic lack of any publicly available prognostic tool that can accurately predict mortality among the general inpatient population.^24^

Machine learning offers a possible solution, with the capability of generating highly accurate mortality predictions using large-volume electronic health record (EHR) data that would otherwise be unaccounted for or underused in mortality risk models. The potential of machine learning predictions to expand SIC has been trialed at a handful of academic hospitals in recent years, with studies showing challenging feasibility but likely improved clinical outcomes. However, firm conclusions have been limited by the absence of controls and the recent variability in health care and patient mix caused by the COVID-19 pandemic.^25,26,27,28,29,30^ We have recently published the structure of an accurate machine learning algorithm for predicting short-term mortality in a modern inpatient population.^31^ By implementing this algorithm in a community hospital with propensity-matched controls, we sought to investigate whether a mortality risk-targeted EHR prompt was associated with increased inpatient goals of care documentation.

The objective of this study was to encourage GOCDs in a community hospital setting with patients identified as having a high risk of dying or entering hospice within 30 days by a machine learning mortality prediction algorithm. We examined how informing physicians of a patient’s high risk of mortality, as identified by a machine learning mortality prediction algorithm, could change the likelihood of documented GOCD before discharge. We hypothesized that physicians who have knowledge of the high risk of mortality in patients will initiate GOCDs prior to discharge.

## Methods

This cohort study was approved by the institutional review board at Washington University in St Louis, and the need for informed consent was waived because patient data were deidentified. This study follows the Strengthening the Reporting of Observational Studies in Epidemiology (STROBE) reporting guideline for cohort studies.

### Cohort and Study Design

This study is a retrospective, difference-in-difference–matched cohort study using EHR data from 4 community hospitals within 1 health care system. The services at each hospital are independent from each other and do not have dedicated resident house staff or teaching services. All hospitals have access to palliative care consultation. The intervention took place at 1 community hospital (Hospital I), a 425-bed community hospital with approximately 19 000 annual admissions in St Louis, Missouri. Control patients were drawn from the 3 other community hospitals within a 25-mile radius of Hospital I in the St Louis area with a combined 412 beds and approximately 22 000 annual admissions.

### Patient Selection

Patients aged 18 years and older who were admitted to hospitalist teams at Hospital I were screened for enrollment from January 2 to July 15, 2021 (Figure). We previously described a machine learning model that ran on the patient’s second hospital day to estimate risk of inpatient mortality, 30-day mortality, and/or hospice discharge.^31^ This model ran on weekdays for all patients admitted within the last 24 to 48 hours, and on Mondays, the model would also include patients admitted up to 72 hours prior. A research team member screened each patient’s medical record on the same day their risk score was calculated. Inclusion criteria included patients on a hospitalist service with a risk score greater than 0.25. This threshold was selected based on preliminary data showing that patients above this risk score had an approximately 6.8-fold increase in the composite mortality or hospice outcome compared with patients with a risk score below 0.25. Up to 4 patients were enrolled in the intervention each day, with priority given to patients with higher risk scores. Race data were taken from hospital registration information and included the categories of Black patients, White patients, and other (ie, American Indian or Alaska Native, Asian, Native Hawaiian or other Pacific Islander, and all other races). Race was considered in this study because previous studies have shown that ACP participation rates differ among racial and ethnic groups.

**Figure.**
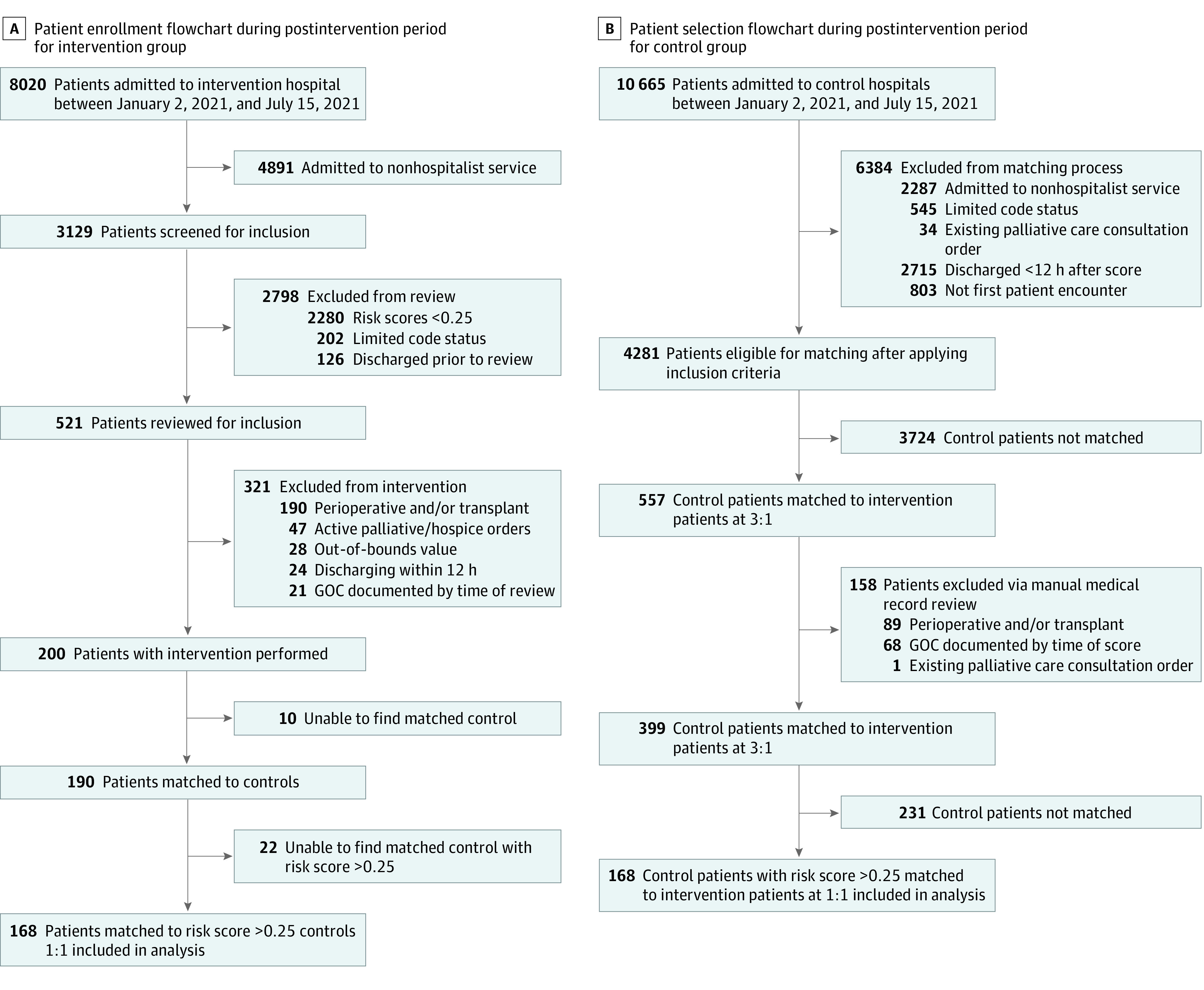
Patient Enrollment Flowchart GOC indicates goal of care.

Exclusion criteria included prior documented ACP or GOCDs, documented limitations in code status, active palliative care or hospice consult orders, patients in the ICU, perioperative patients, solid organ transplantation within the last year, previous enrollment in this study, or physician-documented anticipated discharge within the next 12 hours. Transplant patients were excluded due to the unique clinical decision-making that goes into their care and specifically whether to withdraw care. None of the community hospitals have primary transplant services; any decision to limit care would almost certainly be deferred until the patients were transferred to an academic transplant center. Perioperative patients were also excluded because these patients have these conversations as part of their surgical plan.

During the postintervention period, a retrospective propensity-matched control group was generated from medicine admissions at 3 neighboring community hospitals within the same health care system. A total of 10 665 admissions were included in the potential control sample. Patients aged 18 years and older admitted to hospitalist services for more than 24 hours with risk scores greater than 0.25 and without documented limitations in code status were eligible for matching. Propensity score matching was performed with a nearest neighbor within-caliper match, propensity scores defined between 0 and 1, and a 0.20 caliper on the following variables—age, sex, race, COVID-19 status, and multiple comorbidities.

Due to the need for manual review of inclusion and exclusion criteria, a preliminary control pool was first matched at a ratio of up to 1:3 using Charlson comorbidities in place of risk scores to minimize between-hospital patient population heterogeneity.^32^ After retrospectively reviewing the medical records of this control pool and removing patients who did not meet the same inclusion and exclusion criteria applied to the intervention group, a 1:1 propensity score match was performed using mortality risk scores to generate the final study period control group. The 168 matched pairs were included in the analysis for a poststudy period. To account for temporal heterogeneity and intrinsic hospital differences in ACP and goals of care conversation practices, additional patient cohorts were then generated for a preintervention period sample. In this study, 8429 admissions to the intervention hospital and 10 202 admissions to the control hospitals from June 1 to November 30, 2020, were included in the potential samples. Preintervention period patients at the intervention hospital were matched against intervention group patients in the postintervention period, and preintervention period patients at the 3 control hospitals were matched against the postintervention period control group at a 1:1 ratio using age, sex, race, COVID-19 status, mortality risk score, and hospital. Exact matching was used with sex, race, COVID-19 status, and hospital, and propensity score matching with nearest neighbor within-caliper match was used for mortality risk score with 0.20 caliper. Inclusion and exclusion criteria were applied after matching through manual medical record review. In this study, 94 patients from TGI intervention hospitals and 107 patients from control hospitals were included in the analysis for the preintervention period.

### Study Procedures Measures and Outcomes

For patients in the intervention group, a member of the study team contacted the physician in the morning via secure EHR chat alerting them of the patient’s elevated risk score. Messages were standardized with multiple choice responses encouraging serious illness conversations.

A study team member collected process and outcome data for each patient’s hospitalization. Sociodemographic data were collected from the EHR, as well as medical comorbidities on admission as defined by the Charlson score. The primary outcome was the percentage change of documented GOCD from preintervention to postintervention period during the index hospitalization. GOCDs were defined as any documentation explicitly describing discussion of code status, goals of care, or end-of-life planning. A clinical study team member collected this outcome through manual review of each patient medical record using a combination of search terms including *code status*, *advance care planning*, *palliative care*, and *hospice*, as well as EHR filters to identify notes pertaining to ACP or goals of care. Physician-patient discussions needed to be clearly documented either through free text or an ACP-specific template such as that provided by the study team; statements of code status alone (eg, *code status: full code*) were not sufficient.

A subgroup analysis of documented GOCD per intervention period by race was performed. Secondary outcomes included GOCD free survival, hospital length of stay, discharge code status, palliative care and hospice utilization, in-hospital and 30-day mortality, 30-day ER visits, and 30-day readmission within the health care system. The national Vizient clinical database was used to estimate expected the hospital length of stay. The length of stay indices were adjusted using per-hospital baselines for 2021.

### Statistical Analysis

Propensity scores were estimated through the logistic regression models (Table 1 and eTable 1 in Supplement 1) The standardized mean differences (SMDs) between 2 cohorts were provided for all the interested variables. We considered variables with SMD below 0.1 to be adequately balanced.^33,34^ GOCD free survival was defined as the days from hospital admission to date of documented GOCD. Patients without GOCD were censored at discharge. GOCD outcomes between 2 groups per intervention period were compared using Fisher exact tests for categorical variables, Wilcoxon tests for continuous variables due to departure from normality, and log-rank tests for GOCD free survival. The same statistical methods were used for our main analysis and subgroup analysis (Table 2 and eTable 3 in Supplement 1).

**Table 1.** Patient Demographics and Baseline Characteristics by Intervention Period

Variable	Preintervention period			Postintervention period		
	Control (n = 107)	Intervention (n = 94)	Absolute standardized difference	Control (n = 168)	Intervention (n = 168)	Absolute standardized difference
Mortality risk score						
Mean (SD)	0.44 (0.16)	0.41 (0.14)	0.16	0.44 (0.15)	0.44 (0.17)	0.008
Median (range)	0.39 (0.25-0.85)	0.35 (0.25-0.77)		0.40 (0.25-0.87)	0.39 (0.25-0.91)	
Age, y						
Mean (SD)	76.3 (10.4)	77.2 (10.2)	0.08	79.6 (9.2)	79.3 (9.6)	0.03
Median (range)	76.0 (46.0-97.0)	77.0 (47.0-96.0)		79.5 (51.0-97.0)	81.0 (51.0-98.0)	
Race, No. (%)						
Black	18 (17)	14 (15)	0.02	21 (12)	20 (12)	0.006
White	89 (83)	80 (85)	0.02	144 (86)	145 (86)	0.006
Other^a^	0	0	0	3 (2)	3 (2)	0
Sex						
Female	49 (46)	52 (55)	0.10	85 (51)	85 (51)	<0.001
Male	58 (54)	42 (45)		83 (49)	83 (49)	
COVID-19 status, No. (%)						
Negative	93 (87)	86 (91)	0.05	147 (88)	150 (89)	0.02
Positive	14 (13)	8 (9)		21 (12)	18 (11)	
Charlson Score at discharge						
Mean (SD)	7.20 (3.12)	7.19 (2.80)	0.002	8.81 (3.16)	7.81 (2.63)	0.34
Median (range)	7.00 (1.00-14.0)	7.00 (1.00-16.0)		9.0 (2.0-19.0)	8.0 (2.0-15.0)	

^a^ Other indicates American Indian or Alaska Native, Asian, Native Hawaiian or other Pacific Islander, and all other races.

**Table 2.** Goals of Care Outcomes by Intervention Period

Characteristics	Preintervention period, No. (%)			Postintervention period, No. (%)		
	Control (n = 107)	Intervention (n = 94)	*P* value^a^	Control (n = 168)	Intervention (n = 168)	*P* value^a^
Documented GOCD, No. (%)						
No	95 (89)	79 (84)	.41	141 (84)	68 (40)	<.001
Yes	12 (11)	15 (16)		27 (16)	100 (60)	
GOCD free survival						
Median (95% CI), d	NA (9-NA)	NA (11-NA)	.40	16 (15-NA)	4 (3-5)	<.001
Palliative care notes, No. (%)						
≥1	1 (1)	5 (5)	.10	6 (4)	49 (29)	<.001
None	106 (99)	87 (95)		162 (96)	119 (71)	
Missing	0	2 (2.1)				
Discharge code status, No. (%)						
Comfort care/hospice	0	3 (3)	.08	4 (2)	3 (2)	.002
Full code	102 (95)	83 (88)		152 (90)	132 (79)	
Limited code/DNR	5 (5)	8 (9)		12 (7)	33 (20)	
Hospital LOS						
Mean (SD)	5.46 (3.59)	5.79 (4.08)	.78	7.18 (6.04)	5.97 (3.84)	.10
Median (range)	4.00 (2.00-16.00)	4.00 (1.00-19.00)		5.00 (2.00-39.00)	5.00 (1.00-23.00)	
Vizient LOS index						
Mean (SD)	1.23 (0.93)	0.93 (0.60)	.006	1.20 (0.73)	0.94 (0.63)	<.001
Median (range)	0.95 (0.17-5.61)	0.74 (0.12-3.71)		1.01 (0.25-4.98)	0.75 (0.18-4.60)	
Missing, No. (%)	2 (1.9)	1 (1.1)		0 (0)	2 (1.2)	
Adjusted LOS Index						
Mean (SD)	1.25 (0.94)	1.01 (0.65)	.04	1.22 (0.718)	1.03 (0.69)	<.001
Median (range)	0.98 (0.16-5.32)	0.81 (0.13-4.05)		1.06 (0.23-4.73)	0.82 (0.19-5.02)	
Missing, No. (%)	2 (1.9)	1 (1.1)		0 (0)	2 (1.2)	
Death within 30 d of admission, No. (%)						
No	103 (96)	89 (95)	.74	150 (89)	157 (93)	.24
Yes	4 (4)	5 (5)		18 (11)	11 (7)	
30-d Readmission, No. (%)						
No	88 (82)	85 (90)	.11	143 (85)	139 (83)	.66
Yes	19 (18)	9 (10)		25 (15)	29 (17)	
30-d ED visit, No. (%)						
No	80 (75)	80 (85)	.08	140 (83)	136 (81)	.67
Yes	27 (25)	14 (15)		28 (17)	32 (19)	

Abbreviation: ED, emergency department; DNR, do not resuscitate; GOCD, goals of care discussion; LOS, length of stay; NA, not applicable where event occurrence does not reach 50%.

^a^ Fisher exact tests for categorical variables, Wilcoxon tests for continuous variables, and Log-rank test for GOCD free survival.

Difference-in-difference approaches were used to evaluate changes of advance care planning outcomes between patients attributed to intervention and control hospitals. The linear, logistic, and Cox regression models included the group indicator (intervention vs control), period (postintervention vs preintervention), and the interaction term between group indicator and period. The coefficient estimates and standard error from the linear regression models, odds ratio (OR) and 95% CI from the logistic regression models, and hazard ratio (HR) and 95% CI were presented for the interaction term. We considered hypothesis test results with *P* < .05 statistically significant, and tests were 2-sided. Missing data were handled as missing at random. Demographics and baseline characteristics of patients with complete and missing data for the length of stay index were compared in eTable 2 in Supplement 1. All analyses were performed using R version 1.3.1073 (R Project for Statistical Computing).

## Results

The intervention and control groups during both the preintervention and postintervention period were well-balanced. The preintervention analysis included 94 patients from the intervention hospital (mean [SD] age, 76.3 [10.4] years; 52 [55%] female; 42 [45%] male; 80 [85%] White patients; 14 [15%] Black patients) and 107 patients from control hospitals (mean [SD] age, 76.3 [10.4] years; 49 [46%] female; 58 [54%] male; 89 [83%] White patients; 18 [17%] Black patients). There were then 168 matched controls during the postintervention period. All of these patients were included in the analysis (Figure). The intervention and control groups during the postintervention period were well-balanced with age (mean [SD] age, 79.3 [9.60] years vs 79.6 [9.21] years; SMD, 0.03), sex (female, 85 [51%] vs 85 [51%]; SMD, 0), race (White patients, 145 [86%] vs 144 [86%]; SMD, 0.006; Black patients, 20 [12%] vs 21 [12%]; SMD, 0.006), and Charlson comorbidities (median [range], 8.00 [2.00] vs 9.00 [15.0]; SMD, 0.34). Thirty-nine (11.6%) of the study population were positive for COVID-19 during their admission. Preintervention cohorts were similar between the 2 groups in age (mean [SD] age, 77.2 [10.2] years vs 76.3 [10.4] years; SMD, 0.08), sex (female, 52 [55%] vs 49 [46%]; SMD, 0.10), and race (White patients, 80 [85%] vs 89 [83%]; SMD, 0.02; Black patients, 14 [15%] vs 18 [17%]; SMD, 0.02), and Charlson comorbidities (median [range], 7.00 [1.00-14.0] vs 7.00 [1.00-16.0]; SMD, 0.002) (Table 1).

Rates of preintervention period GOCD documentation (15 [16%] vs 12 [11%]; *P* = .41), palliative care consultation (none, 87 [95%] vs 106 [99%]; ≥1 note, 5 [5%] vs 1 [1%]; *P* = .10), and code status deescalation (full code, 83 [88%] vs 102 [95%]; limited code or do not resuscitate, 8 [9%] vs 5 [5%]; comfort care or hospice, 3 [3%] vs 0; *P* = .08) and GOCD free survival (median [range], not applicable [NA] [11-NA] vs NA [9-NA]; *P* = .40) in these mortality risk-matched groups were not significantly different between intervention and control hospitals (Table 2). In the postintervention period, there were significantly higher rates of GOCD documentation (100 [60%] vs 27 [16%]; *P* < .001), palliative care consultation (≥1 note, 49 [29%] vs 6 [4%]; *P* < .001), and code status deescalation (35 [22%] vs 16 [9%], *P* = .002) in the intervention group (Table 2). GOCD-free survival analysis demonstrated that GOCD occurred earlier in the hospitalization for intervention patients compared with the control group (median, 4 days; 95% CI, 3-5 vs 16 days; 95% CI, 15-NA; *P* < .001). There were no statistically significant differences in other secondary outcomes (Table 2).

Subgroup analysis by race in the postintervention period (eTable 3 in Supplement 1) demonstrated similar findings of increased GOCD in both Black patients (70% vs 33%; *P* = .03) and White patients (57% vs 14%; *P* < .001). GOCD also occurred earlier in the hospitalization compared with the control group in both Black patients (median 4 days; 95% CI, 3-NA vs 22 days; 95% CI, 6-NA, *P* = .001) and White patients (median 4 days; 95% CI, 3-6 vs 16 days, 95% CI, 15-NA, *P* < .001).

Difference-in-difference analysis between the postintervention period and preintervention period cohorts showed intervention patients from preintervention to postintervention period were significantly more likely to have documented GOCDs (OR, 5.11; 95% CI, 1.93-13.42; *P* = .001) (Table 3). GOCD free survival also indicated higher GOCD (HR, 4.69; 95% CI, 1.95-11.27; *P* < .001) from pre- to postintervention period for intervention patients when compared with matched controls. Other secondary outcomes were not statistically significantly different between the preintervention and postintervention cohorts.

**Table 3.** Changes From Preintervention to Postintervention Period in the Outcomes Relative to Control

Outcome	Difference-in-difference estimate (SE)^a^	*P* value	OR (95% CI)	Absolute change (95% CI)	
				Control	Intervention
Documented GOCD	1.63 (0.49)	.001	5.11 (1.93 to 13.42)	NA	NA
GOCD free survival	1.55 (0.45)	<.001	4.69 (1.95 to 11.27)^b^	NA	NA
Palliative care notes	0.60 (1.19)	.61	1.83 (0.09 to 14.70)	NA	NA
Hospital LOS, d	−1.54 (0.83)	.06	NA	1.72 (0.58 to 2.86)	0.18 (−0.83 to 1.20)
Vizient LOS index	0.04 (0.13)	.78	NA	−0.03 (−0.24 to 0.18)	0.01 (−0.14 to 0.17)
Adjusted LOS index	0.05 (0.13)	.73	NA	−0.03 (−0.25 to 0.18)	0.02 (−0.16 to 0.18)
Death within 30 d of admission	−0.91 (0.79)	.25	0.40 (0.08 to 1.92)	NA	NA
30-d Readmission	0.89 (0.52)	.09	2.43 (0.89 to 7.02)	NA	NA
30-d ED visit	0.82 (0.46)	.08	2.27 (0.92 to 5.71)	NA	NA
Change in code status	0.04 (0.65)	.95	1.04 (0.30 to 3.95)	NA	NA
Hospice referral	−0.79 (0.72)	.28	0.45 (0.11 to 1.89)	NA	NA
Hospice enrollment	0.58 (0.96)	.54	1.80 (0.30 to 14.87)	NA	NA

Abbreviations: ED, emergency department; GOCD, goals of care discussion; HR, hazards ratio; LOS, length of stay; NA, not applicable; OR, odds ratio.

^a^ Estimates from linear, logistic, or Cox models depending on outcome.

^b^ Data reported as HR (95% CI).

## Discussion

In this propensity score-matched cohort study, we informed physicians of patients with a high risk of mortality during the next 30 days to facilitate GOCDs. Compared with propensity-matched controls, physicians who received messages were 5 times more likely to have GOCDs with patients prior to discharge than those who did not receive mortality information, and the GOCDs occurred significantly earlier in the hospitalization. These findings suggest that a risk-based message to physicians is associated with a greater likelihood of engaging in GOCDs in community hospital settings.

The integration of mortality risk into clinical practice remains a subject of ongoing investigation. Several academic hospitals have recently published promising workflows and initiatives to enhance serious illness conversations,^35,36,37,38^ including a recent study of 20 506 oncology patients using a machine-learning algorithm to generate prompts, which successfully increased serious illness conversations from 3.4% to 13.5% and decreased end-of-life systemic therapy.^39^ In these studies, multiple barriers have been identified, including lack of clinician engagement, alert fatigue, restricted or biased prediction models, and limited practice capacity for serious illness conversations.^40,41,42^ Our study offers an example of how these challenges can be partially attenuated while also demonstrating that mortality risk-based predictions can be applied in a community setting, which has been previously highlighted as a need.^43^ Clinician fatigue was mitigated by a combination of manual medical record review to ensure the appropriate patient selection and targeted messages in lieu of pop-up or click-through alerts. Our mortality model was shown to apply broadly to all inpatients, even in the modern COVID-19 era, and our palliative care team was closely involved in our implementation to ensure sufficient resources were readily available.

Another notable finding is that our pilot intervention was associated with similar improvements in early GOCD in both Black patients and White patients. This result is consistent with subgroup analysis from our machine learning model development, in which we showed that our model accurately predicted mortality in both Black patients and White patients in contrast to many traditional mortality scores. Considering that several past studies have shown significant disparities in engaging Black patients in GOCDs, this suggests that machine learning may play a pivotal role in improving access to such discussions in minorities.^44,45^ However, given our limited subgroup size, future research on this crucial topic will be required.

### Study Limitations

This study had limitations. First, our mortality model was developed using data from a single health care system, and our study intervention was implemented at a single community hospital within that system. While we believe our mortality model structure may be readily replicated at other institutions, factors, such as the EHR, clinical practice, and population heterogeneity, may limit its applicability to other health care systems. Second, the potential confounding of the COVID-19 pandemic throughout our study cannot be overstated. While we attempted to reduce the magnitude of this effect by choosing geographically colocated hospitals and including COVID-19 status as a matching variable, there were innumerable pandemic-related changes in health care delivery at each site that may have influenced our results. For example, palliative care consultations at 1 control site were relegated to telemedicine visits for a portion of the study period, which may be associated with altered practice patterns. Third, this pilot study was not powered to detect differences in clinical outcomes, and the small sample size dictates that our findings should be considered exploratory in nature. Fourth, the intervention was implemented without training the physicians on serious illness conversation and goals of care communication skills. The association may have been greater with appropriate training beforehand. Despite these limitations, the study provides promising results and identifies opportunities for future research. Fifth, we used relatively restrictive patient selection criteria. By excluding perioperative, transplant, and patients in the ICU, as well as patients with preexisting limitations on code status and palliative care orders placed within the first 24 hours of admission, we effectively removed the most moribund patients from our potential study population. Whether these high-risk patients would stand to benefit from a goals of care intervention remains to be seen. Despite these limitations, the findings suggest a promising path forward to improving serious illness conversations, specifically GOCDs.

## Conclusions

The findings of this study suggest that a clinical intervention using machine learning mortality predictions was associated with significant increases in GOCDs among high-risk medical inpatients, which occurred earlier in the hospital course. The next steps include the need for an intervention randomized trial in multiple sites to improve generalizability and assess the customization needs across hospital systems.

## Notes

Supplement 1. **eTable 1.** Patient Demographics and Baseline Characteristics by Group **eTable 2.** Comparison of Patients with Missing and Nonmissing Data for LOS Index by Intervention Period **eTable 3.** GOCD Subgroup Analysis by Race and Intervention Period

Supplement 2.
Data Sharing Statement
