# Supplementary material for: Advanced Care Planning for Hospitalized Patients Following Clinician Notification of Patient Mortality by a Machine Learning Algorithm
Source: JAMA Netw Open. 2023 Apr 18;6(4):e238795. doi: 10.1001/jamanetworkopen.2023.8795 (PMC10114011; doi:10.1001/jamanetworkopen.2023.8795)
Supplement: Supplement 1. — eTable 1. Patient Demographics and Baseline Characteristics by Group eTable 2. Comparison of Patients with Missing and Nonmissing Data for LOS Index by Intervention Period eTable 3. GOCD Subgroup Analysis by Race and Intervention Period [file jamanetwopen-e238795-s001.pdf]

Supplemental Online Content

Chi S, Kim S, Reuter M, et al. Advanced care planning for hospitalized patients following clinician notification of patient mortality by a machine learning algorithm. *JAMA Netw Open*. 2023;6(4):e238795. doi:10.1001/jamanetworkopen.2023.8795

eTable 1. Patient Demographics and Baseline Characteristics by Group

eTable 2. Comparison of Patients with Missing and Nonmissing Data for LOS Index by Intervention Period

eTable 3. GOCD Subgroup Analysis by Race and Intervention Period

This supplemental material has been provided by the authors to give readers additional information about their work.

© 2023 Chi S et al. *JAMA Network Open.*

eTable 1. Patient Demographics and Baseline Characteristics by Group

	Control			Intervention		
Variable	Pre-Intervention Period (N=107)	Post-Intervention Period (N=168)	Standardized Difference (abs)	Pre-Intervention Period (N=94)	Post-Intervention Period (N=168)	Standardized Difference (abs)
**Mortality Risk Score**						
Mean (SD)	0.437 (0.160)	0.438 (0.149)	0.007	0.414 (0.141)	0.437 (0.165)	0.151
Median [Min, Max]	0.389 [0.250, 0.845]	0.396 [0.251, 0.868]		0.354 [0.254, 0.765]	0.388 [0.252, 0.906]	
**Age**						
Mean (SD)	76.3 (10.4)	79.6 (9.21)	0.336	77.2 (10.2)	79.3 (9.60)	0.216
Median [Min, Max]	76.0 [46.0, 97.0]	79.5 [51.0, 97.0]		77.0 [47.0, 96.0]	81.0 [51.0, 98.0]	
**Race**						
White	89 (83%)	144 (86%)	0.025	80 (85%)	145 (86%)	0.012
Black	18 (17%)	21 (12%)	0.043	14 (15%)	20 (12%)	0.030
Other	0 (0%)	3 (2%)	0.018	0 (0%)	3 (2%)	0.018
**Gender**						
Female	49 (46%)	85 (51%)	0.048	52 (55%)	85 (51%)	0.047
Male	58 (54%)	83 (49%)		42 (45%)	83 (49%)	
**COVID Status**						
COVID-	93 (87%)	147 (88%)	0.006	86 (91%)	150 (89%)	0.022
COVID+	14 (13%)	21 (12%)		8 (9%)	18 (11%)	
**Charlson Score at Discharge**						
Mean (SD)	7.20 (3.12)	8.81 (3.16)	0.514	7.19 (2.80)	7.81 (2.63)	0.228
Median [Min, Max]	7.00 [1.00, 14.0]	9.00 [2.00, 19.0]		7.00 [1.00, 16.0]	8.00 [2.00, 15.0]	

© 2023 Chi S et al. *JAMA Network Open.*

eTable 2. Comparison of Patients with Missing and Nonmissing Data for LOS Index by Intervention Period

	Pre-Intervention Period			Post-Intervention Period		
	Missing (N=3)	Not Missing (N=198)	P-value[1]	Missing (N=2)	Not Missing (N=334)	P-value[1]
**Mortality Risk Score**						
Mean (SD)	0.459 (0.212)	0.426 (0.151)	0.745	0.402 (0.0113)	0.438 (0.157)	0.858
Median [Min, Max]	0.353 [0.320, 0.702]	0.371 [0.250, 0.845]		0.402 [0.394, 0.410]	0.393 [0.251, 0.906]	
**Age (years)**						
Mean (SD)	71.7 (8.39)	76.8 (10.3)	0.344	71.0 (7.07)	79.5 (9.39)	0.173
Median [Min, Max]	76.0 [62.0, 77.0]	77.0 [46.0, 97.0]		71.0 [66.0, 76.0]	80.0 [51.0, 98.0]	
**Race**						
White	2 (67%)	167 (84%)	0.407	1 (50%)	40 (12%)	0.261
Black	1 (33%)	31 (16%)		1 (50%)	288 (86%)	
Other	0 (0%)	0 (0%)		0 (0%)	6 (2%)	
**Gender**						
Female	1 (33%)	100 (51%)	0.621	2 (100%)	164 (49%)	0.243
Male	2 (67%)	98 (49%)		0 (0%)	170 (51%)	
**COVID Status**						
COVID-	3 (100%)	176 (89%)	1	2 (100%)	295 (88%)	1
COVID+	0 (0%)	22 (11%)		0 (0%)	39 (12%)	
**Charlson Score at Discharge**						
Mean (SD)	7.67 (4.04)	7.19 (2.96)	0.880	11.0 (2.83)	8.29 (2.95)	0.183
Median [Min, Max]	7.00 [4.00, 12.0]	7.00 [1.00, 16.0]		11.0 [9.00, 13.0]	8.00 [2.00, 19.0]	

[1]Fisher exact tests for categorical variables and Wilcoxon tests for continuous variables.

© 2023 Chi S et al. *JAMA Network Open.*

eTable 3. GOCD Subgroup Analysis by Race and Intervention Period

	Pre-Intervention Period			Post-Intervention Period		
Black Patients	Control (N=18)	Intervention (N=14)	P-value[1]	Control (N=21)	Intervention (N=20)	P-value[1]
**Documented GOCD**			0.113			0.029
No	14 (78%)	14 (100%)		14 (67%)	6 (30%)	
Yes	4 (22%)	0 (0%)		7 (33%)	14 (70%)	
**GOCD free survival**			0.090			0.001
Median (95% CI, *in days*)	9 (7, NA)	NA (NA, NA)		22 (6, NA)	4 (3, NA)	
White Patients	Control (N=89)	Intervention (N=80)		Control (N=144)	Intervention (N=145)	
**Documented GOCD**			0.075			<0.001
No	81 (91%)	65 (81%)		124 (86%)	62 (43%)	
Yes	8 (9%)	15 (19%)		20 (14%)	83 (57%)	
**GOCD free survival**			0.090			<0.001
Median (95% CI, *in days*)	NA (NA, NA)	NA (11, NA)		16 (15, NA)	4 (3, 6)	

[1]Fisher exact test for categorical variable and Log-rank test for GOCD free survival. NA: Not applicable where event occurrence does not reach 50%.

© 2023 Chi S et al. *JAMA Network Open.*
